# Pedicle subtraction osteotomy and disc resection with cage placement in post-traumatic thoracolumbar kyphosis, a retrospective study

**DOI:** 10.1186/s13018-016-0447-1

**Published:** 2016-10-12

**Authors:** Wenhao Hu, Bin Wang, Hongyu Run, Xuesong Zhang, Yan Wang

**Affiliations:** 1 The Department of Orthopaedics, Chinese PLA General Hospital, Beijing, People’s Republic of China; 2 Qianxi County People’s Hospital, Wall Road, Qianxi County, Hebei Province People’s Republic of China

**Keywords:** Pedicle subtraction osteotomy, Disc resection, Cage placement, Long-segment fixation, Pelvic tilt, Sagittal vertical axis

## Abstract

**Background:**

It is estimated that upwards of 50,000 individuals suffer traumatic fracture of the spine each year, and the instability of the fractured vertebra and/or the local deformity results in pain and, if kyphosis increases, neurological impairment can occur. There is a significant controversy over the ideal management. The purpose of the study is to present clinical and radiographic results of pedicle subtraction osteotomy and disc resection with cage placement in correcting post-traumatic thoracolumbar kyphosis.

**Methods:**

From May 2010 to May 2013, 46 consecutive patients experiencing post-traumatic thoracolumbar kyphosis underwent the technique of one-stage pedicle subtraction osteotomy and disc resection with cage placement and long-segment fixation. Pelvic incidence (PI), pelvic tilt (PT), sagittal vertical axis (SVA), and sagittal Cobb angle were measured to evaluate the sagittal balance. Oswestry disability index (ODI), visual analog scale (VAS), and general complications were recorded.

**Results:**

The average surgical time was 260 min (240–320 min). The mean intraoperative blood loss was 643 ml (400–1200 ml). The maximum correction angle was 58° with an average of 47°, and the SVA improved from +10.7 ± 3.5 cm (+7.2 to + 17.1 cm) to +4.1 ± 2.7 cm (+3.2 to + 7.6 cm) at final follow-up (*p* < 0.01). PT reduced from preoperative 27.2 ± 5.3° to postoperative 15.2 ± 4.7° (*p* < 0.01). The VAS changed from preoperative 7.8 ± 1.6 (5.0–9.0) to 3.2 ± 1.8 (2.0–5.0) (*p* < 0.01). Clinical symptoms and neurological function were significantly improved at the final follow-up. All patients completed follow-up of 41 months on average.

**Conclusions:**

Pedicle subtraction osteotomy and disc resection with cage placement and long-segment fixation are effective and safe methods to treat thoracolumbar post-traumatic kyphosis.

## Background

It is estimated that upwards of 50,000 individuals suffer traumatic fracture of the spine each year [1], and a large proportion of traumatic thoracolumbar fracture can present a regional kyphotic deformity due to inappropriate treatment methods or time delay. Pathological changes in post-traumatic kyphosis (PTK) include muscle or disc degeneration, canal or neuroforamen compromise, and local instability [2]. The instability of the fractured vertebra and/or the local deformity results in pain and, if kyphosis increases, neurological impairment can occur [3]. Surgical treatment is aimed at correcting the kyphosis and spinal cord decompression, decreasing pain, and improving neurologic function [4]. Post-traumatic thoracolumbar kyphosis presents several challenges to the spine surgeon.

Since Smith-Peterson osteotomy technique was firstly described in 1945, several procedures have been proposed to correct kyphosis. Anterior, posterior, or anterior combined with posterior approaches show various degrees of success for correcting thoracolumbar kyphosis. The anterior approach usually involves a longer operation time and may injure the internal organs, in addition to the possibility of the prosthesis sinking into an osteoporotic spine [5]. Currently, the posterior approach is the most common treatment, and pedicle subtraction osteotomy (PSO) was introduced several years ago as an alternative method with favorable outcome [6–8]. However, the surgical results are not always satisfactory [9], and there is a significant controversy over the ideal management [10].

In this retrospective cohort study, we modified pedicle subtraction osteotomy combined with cage placement for the correction of thoracolumbar deformity and long-segment fixation was used to restore the normal sagittal alignment and stabilize the spine column. The goal of this paper is to present clinical and radiographic results of patients treated with the technique.

## Methods

From May 2010 to May 2013, 46 consecutive patients (24 males, 22 females; mean age 51.7 years) experiencing post-traumatic thoracolumbar kyphosis underwent one-stage pedicle subtraction osteotomy and disc resection with cage placement at our hospital. This study was conducted with approval from the Ethics Committee of Chinese PLA General Hospital. Written informed consent was obtained from all participants. The average interval between initial fracture and kyphosis correction was 28 months (range, 8–96 months). The level of kyphotic apices was T11 in 7 cases, T12 in 18 cases, L1 in 16 cases, and L2 in 5 cases. Of the 46 patients, 20 accepted surgery at the time of injury, 21 patients with less severe injuries were recommended to non-operative modalities and serial imaging to evaluate bony healing and alignment, and 5 patients refused the operation because of financial issues. The initial surgical treatments included simple laminectomy in 6 patients, laminectomy and short-segment pedicle screw fixation in 11 patients, and posterior open reduction and pedicle screw fixation in 3 patients. Two patients developed deep wound infection at the surgical site, and the implants were removed 1 and 2 months later, respectively. The other 12 patients with instrumentation underwent removal of the implants because of pain and discomfort 12–24 months post-operatively.

The indications for surgery were as follows: (1) the Cobb angle exceeding 30 of sagittal index; (2) significant pain refractory to conservative treatment; and (3) increasing neurological deficit. Conservative treatment including bed rest, immobilization, and pain management program should last for at least 3 months. Patients with obvious rotation of vertebrae and multiple fractures who need two or multi-level spinal osteotomy were not included in this study. Before surgery, 21 patients complained of intractable pain in the thoracolumbar junction regions. Neurological deficits were assessed according to American Spinal Injury Association (ASIA) grading system: ASIA E, 22 cases; ASIA D, 19 cases; ASIA C, 5 cases.

### Surgical procedures

In our center, all surgeries were performed under monitoring of somatosensory-evoked potentials, transcranial motor-evoked potentials, and free-running electromyography. There was no preoperative embolization at the operated level.

**Fig. 1:**
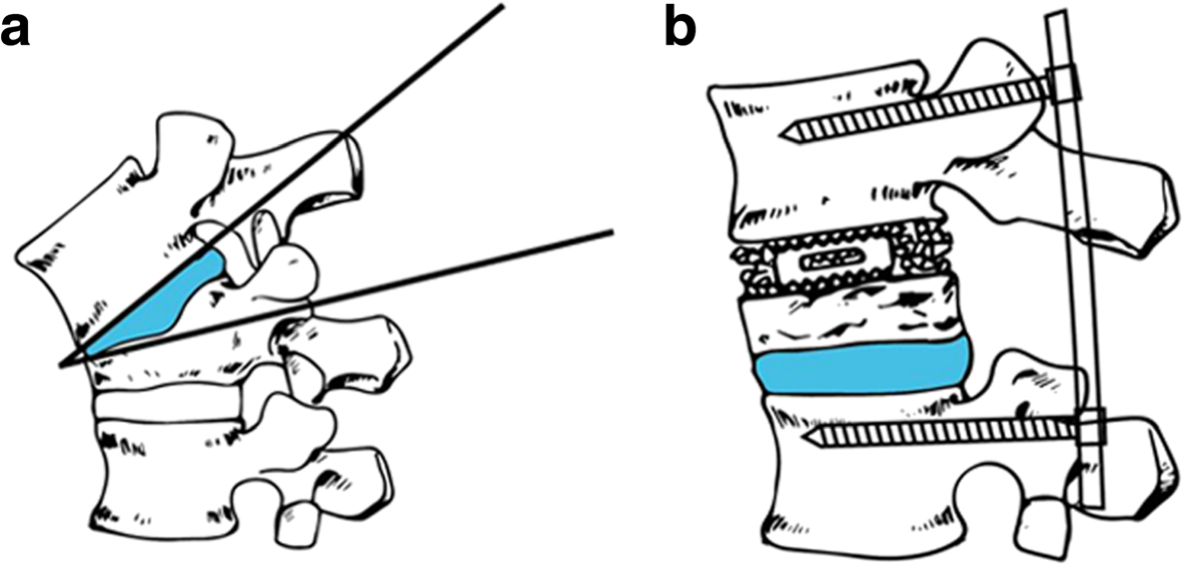
**a** The injured upper disc and posterior elements were removed. **b** A PEEK cage with autogenous bone was inserted into the osteotomy space, effective kyphosis correction was achieved, and the spinal column height was restored

Under general anesthesia, the patient was placed in a prone position on a radiolucent table. The osteotomy sites are usually chosen at the apex of the deformity based on radiographs and clinical evaluation. A posterior midline incision was made to expose the osteotomy site and adjacent three segments above and below. Subperiosteal dissection performed laterally to the transverse processes in the lumbar spine and to the costotransverse junction in the thoracic spine. The segmental vessels were coagulated using electric cauterization and hemostatic gauze. Monoaxial pedicle screws (Weigao Orthopedic, Shandong, China) were then placed three levels above and below the damaged vertebral body by freehand technique. The posterior elements including the spinous process, bilateral lamina, transverse process, and the adjacent facet joints at the level of apical vertebra are needed to be removed. In the thoracic spine, the proximal 3 cm of bilateral ribs and the rib head were also excised. In patients who have undergone initial surgery, laminectomy was performed at a level adjacent to the previous decompression to expose the dura and epidural scarring is carefully released. Next, the osteotomy procedure was performed (Fig. 1), bilateral external walls of the damaged vertebrae are exposed with a sponge stick and periosteal elevator, and two thirds of the upper pedicle was resected with a pedicle probe and drill. A transpedicular decancellous procedure was performed within the target vertebrae with a curette, and the upper one third to one half of the vertebrae was removed. Then, the bilateral cortical bone of the vertebrae is removed with a rongeur to make space for removing the upper disc with Cobb elevator. After the above procedure, the posterior portion of the vertebral body was carefully resected with a reverse angular rongeur. Any adhesions between the dura and bone should be freed. A temporary rod was placed when one side of the osteotomy was completed. From a lateral side, a polyetheretherketone (PEEK) cage (Weigao Orthopedic, Shandong, China), an interbody fusion device, with the bone autografts was inserted into the resection space at the anterior column between the lower endplate of the upper vertebra and the osteotomy surface.

For the correction technique, a precontoured rod was locked down into the inferior screws and the temporary rod was removed, the rod was then cantilevered into the superior screws with gentle manual downward pressure to correct the kyphosis. The contralateral rod was placed, and the entire construct was tightened finally. The posterior interlaminar fusion was done over the fixed segments. After confirmation of absent soft or bony compression, a drainage tube was placed in the surgical field, and the wound was closed in layer sequence.

Post-operatively, the drainage tube was left until the output was less than 50 ml/24 h, usually for 3–5 days, and a custom-made plastic thoracolumbosacral orthosis was used for 3 months.

### Radiologic and clinical evaluation

**Fig. 2:**
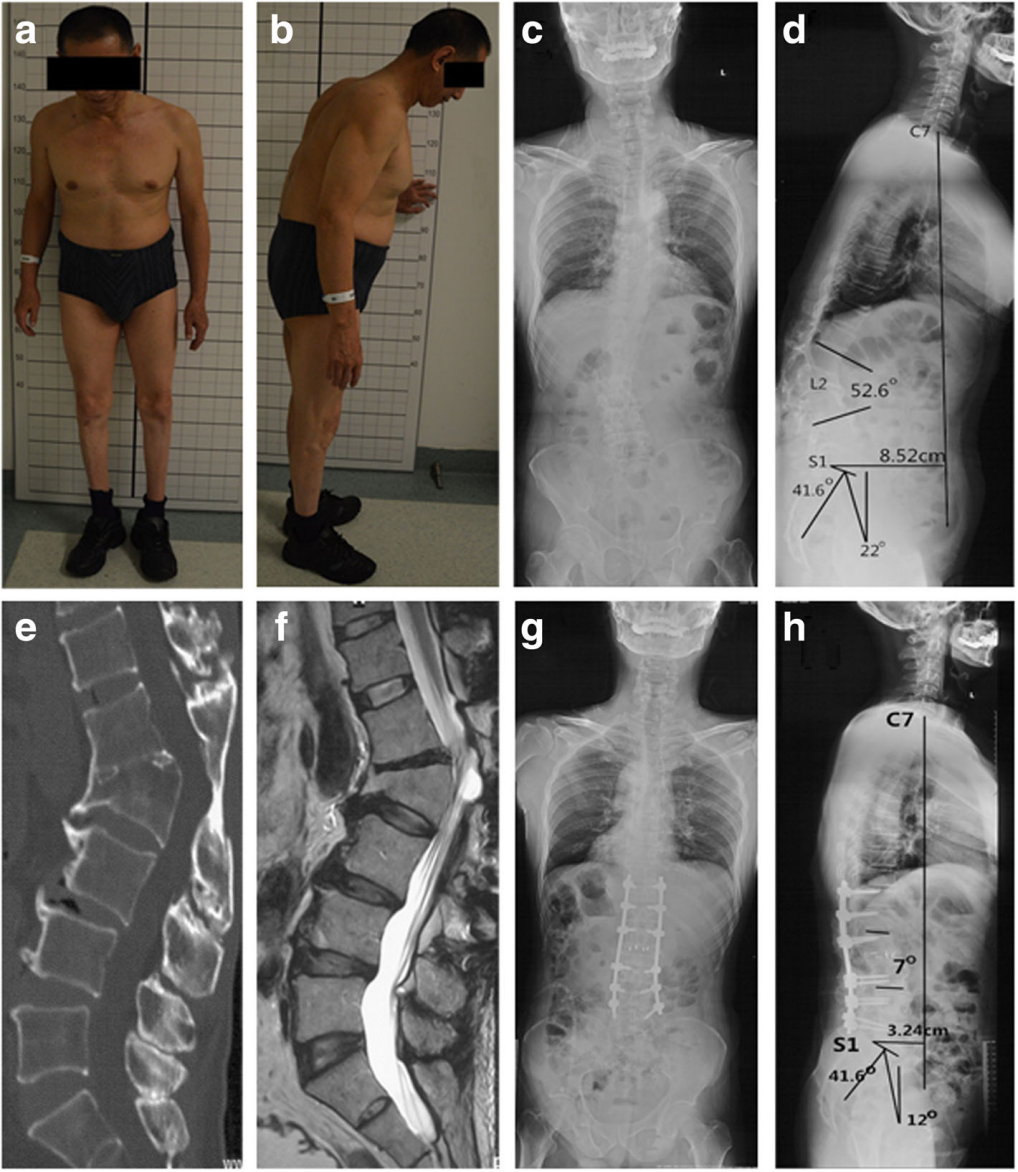
A 55-year-old male patient (**a**, **b**) who is suffering from severe back pain and numbness in the right thigh for more than 12 months due failure of conservative treatment. Preoperative anteroposterior (**c**), lateral radiographs (**d**), and CT (**e**), MRI (**f**) show that the apex of kyphosis is located at L2 with PI 41.6°, PT 22°, SS 20°, SVA 8.52 cm, and the Cobb angle is 52.6°. Three years postoperatively anteroposterior (**g**) and lateral radiographs (**h**) of L2 with a 7 Cobb angle, PT 12°, SS 37°, SVA 3.24 cm, and bony fusion was achieved

Radiographs and clinical evaluation was performed preoperatively, post-operatively, and at the final follow-up. Preoperative and post-operative full-length spine radiographs including the whole spine and pelvis were available for all patients (Fig. 2). Pelvic incidence (PI), pelvic tilt (PT), sacral slope (SS), sagittal vertical axis (SVA) were documented to evaluate the global sagittal balance. The Cobb angle was measured between the upper end plate of the vertebra one above fracture and the lower end plate of the vertebra one below fracture to assess kyphosis deformity on lateral radiograph. Radiologic assessment of fusion at follow-up was based on the presence of the trabecular bone bridging at the osteotomy site as described by Brantigan and Steffee [11]. Clinical outcomes were assessed using Oswestry disability index (ODI), and back pain was measured by visual analog scale (VAS). Surgical time, blood loss, and general complications were recorded (Table 1).

**Table 1:** Summary of preoperative and intraoperative characteristics

Characteristics	
Number of patients	46
Male/female	24/22
Age (year)	51.7 ± 8.6
Time interval (month)	28.4 ± 15.3
Follow-up (month)	41.2 ± 10.4
Surgical time (min)	260.6 ± 47.3
Blood loss (ml)	643.3 ± 221.6

### Statistical analysis

Each variable was presented as mean ± SD. Statistical analyses were performed using paired *t* test (SPSS 19.0, SPSS Inc.) before and after surgery. Normality was assumed and *p* < 0.05 was deemed as significant difference.

## Results

Pedicle subtraction osteotomy and disc resection with cage placement and long-segment fixation were performed in all patients. All patients completed follow-up of 41 months on average, from 34 to 52 months. Dural tears were encountered in two cases, which were successfully repaired with 6-0 prolene suture intraoperatively. No deep wound infection was identified. Two patients suffered transient partial neurological deficit post-operatively, one patient presented a temporary unilateral iliopsoas weakness (grade 4/5) that recovered within 1 months the other patient presented a unilateral quadricipital weakness (grade 4/5) that resolved completely within 2 months. New permanent neurological deficit was absent in our study. The mean ODI was 56.2 % (38.0 to 91.0 %) preoperatively to 28.6 % (14.0 to 52.0 %) at the final follow-up (*p* < 0.01). Neurological function improved from ASIA scale D to E in 14 patients, from ASIA scale C to D in 4 patients.

The sagittal vertical axis (SVA) improved from +10.7 ± 3.5 cm (+7.2 to + 17.1 cm) to +4.1 ± 2.7 cm (+3.2 to + 7.6 cm) (*p* < 0.01) (Table 2). The maximum correction angle was 58° with an average of 47°. Solid fusion was obtained in all patients at the final follow-up according to radiological evidence, and no implant failures were noted.

**Table 2:** Summary of clinical and radiologic outcomes

*n* = 46	Preoperative	Post-operative	*P*
PI (°)	44.1 ± 7.8		
PT (°)	27.2 ± 5.3	15.2 ± 4.7	<0.001
SS (°)	20.2 ± 4.7	31 ± 5.6	<0.001
SVA (cm)	10.7 ± 3.5	4.1 ± 2.7	<0.001
Cobb (°)	49.1 ± 3.4	7.2 ± 3.7	<0.001
ODI (%)	56.2 ± 17.0	28.6 ± 12.6	<0.001
VAS	7.8 ± 1.6	3.2 ± 1.8	<0.001
ASIA			
A(n)	0	0	
B(n)	0	0	
C(n)	5	1	
D(n)	19	9	
E(n)	22	36	

*PI* pelvic incidence, *PT* pelvic tilt, *SS* sacral slope, *ODI* Oswestry disability index, *SVA* sagittal vertical axis, *VAS* visual analog scale, *ASIA* American Spinal Injury Association

## Discussion

Kyphosis deformity following thoracolumbar vertebral fracture is often caused by inappropriate conservative treatment, inadequate immobilization, too early weight-bearing, incorrect surgical procedure and fixation, and improper choice of internal fixation devices [12]. PTK in thoracolumbar junction region may compress the nerve root or impair the blood circulation, which leads to injury of the conus or cauda equina. Patients who were treated inadequately usually experience chronic pain, which may be from the deformity site itself, the injured disc, a bony nonunion, or the lordotic compensation above and below the deformity site where additional stress is placed on the respective facet joints [13]. If hyperlordosis of the lumbar tract occurs, the posterior loading of the articular facets may result in low back pain, even if the fracture is cranial [14]. Post-traumatic kyphosis is usually characterized by fixed and sharp angle, which makes the correction difficult.

Several forms have been taken to correct the deformity. Though the anterior approaches allow direct decompression since the pathology located in the anterior and middle spinal columns, this procedure is time-consuming and may increase the probability of complications such as hemorrhage and pulmonary or gastrointestinal dysfunction. Moreover, one of the shortcomings of the anterior techniques has been reported to be the difficulty to restore the anterior column height to normal, resulting in post-operative persistence of some degree of kyphosis [15].

Suk et al. [16] compared the surgical results between combined AP procedures and posterior closing wedge osteotomy procedures in cases of post-traumatic kyphosis. They believed that the posterior procedure had shorter surgical time and less intraoperative bleeding, diminished post-operative morbidity, and more reliably restored sagittal alignment and provided solid spinal arthrodesis with the application of the transpedicular spinal system under favorable compression conditions.

Currently, the one-stage posterior approach is more popular for correction of post-traumatic thoracolumbar deformity. Pedicle subtraction osteotomy (PSO) is the most commonly recommended technique to correct local post-traumatic thoracolumbar deformity because it does not lengthen the anterior column and potentially reduces the need for an anterior procedure [17]. Wu et al. [6] performed posterior PSO in 13 patients, achieved an average correction of 38.8° with no neural injuries. Yong-Ming Xi et al. [2] used pedicle subtraction osteotomy to obtain an average correction angle of 47°. After vertebral fractures, however, the injured upper level disc may fall into the vertebrae through the collapsed end plate, which results in disc degeneration, secondary kyphosis, or treatment failure [18]. In this situation, solid fusion is difficult to achieve. Rui Gao et al. [9] proposed a modified PSO in which the injured upper end plate and upper-level disc were routinely removed and the inferior wall of the pedicle and the lower facet joint were preserved, the mean correction of focal kyphotic deformity was 34.5°. Zhang et al. [10] described a modified posterior closing wedge osteotomy that the upper disc and facet joint were removed and obtain greater angle osteotomy correction (mean correction angle 42°). The main advantages of taking down the disc are the added amount of correction provided, and the additional fusion surface. With a large interspace, however, the spinal column will be excessively shortened which may result in buckling of the dura and spinal cord. In our study, after removing the upper disc and posterior elements, a cage containing autogenous bone was put at the anterior column between the lower end plate of the upper vertebra and the osteotomy surface to restore the spinal column height and avoid potential spinal cord curving or kinking. Furthermore, the anterior cage could act as a hinge during the closure of osteotomy to avoid the occurrence of sagittal translation and ensures the safety of this procedure. Therefore, the large correction angle was achieved safely with the technique of modified PSO combined with cage placement.

In addition, patients with a sagittal imbalance caused by the kyphosis show a compensatory hyperlordosis of the segments above and/or below the kyphotic segment [19], and when the regional alignment is corrected, the adjacent vertebral diseases may occur. An implant failure has been noted in patients treated with short-segment fixation [20]. Therefore, long-segment fixation is performed in the current study to retrieve the global sagittal balance and stabilize the spine column.

All of the 46 patients with post-traumatic thoracolumbar deformity who underwent pedicle subtraction osteotomy and disc resection with cage placement and long-segment fixation achieved satisfactory rehabilitation and none required revision surgery.

## Conclusions

Pedicle subtraction osteotomy and disc resection with cage placement and long-segment fixation are good choice for post-traumatic kyphosis, which contribute to satisfactory kyphosis deformity correction and global sagittal balance. Our study suggests that these were effective and safe methods to treat thoracolumbar post-traumatic kyphosis.

## Abbreviations

ASIA: American Spinal Injury Association
PI: Pelvic incidence
PSO: Pedicle subtraction osteotomy
PT: Pelvic tilt
PTK: Post-traumatic kyphosis
SS: Sacral slope
SVA: Sagittal vertical axis
VAS: Visual analog scale
